# Diagnosing earth's tipping points: where we stand in the Anthropocene

**DOI:** 10.3389/fpubh.2025.1653860

**Published:** 2025-09-01

**Authors:** Johan Rockström

**Affiliations:** Potsdam Institute for Climate Impact Research (PIK), Potsdam, Germany

**Keywords:** planetary health, climate change, Anthropocene, earth system science, sustainability, tipping points, biosphere integrity, resilience

## Abstract

Human and planetary health are inextricably linked through complex adaptive systems. This perspective, adapted from the 2024 Virchow Lecture, highlights how accelerating anthropogenic pressures are destabilizing the Earth system. Scientific evidence shows that six of nine planetary boundaries have already been transgressed, increasing the risk of irreversible tipping points. The Holocene epoch, a period of climate stability underpinning human civilization, serves as a benchmark for a “safe and just operating space” for humanity. Staying within this space is essential not only for ecological resilience but also for achieving the United Nations Sustainable Development Goals (SDGs), which depend on a stable Earth system. The biosphere's capacity to regulate climate is under severe threat. Without urgent action to restore and protect nature and its natural carbon sinks, even full decarbonization by 2050 may not prevent systemic breakdowns. The Earth still exhibits self-dampening feedbacks, which offer a narrow but critical opportunity to shift course. This article calls for integrated action across climate, health, and sustainability science and introduces the Planetary Health Check as an annual tool to assess Earth's condition. The path to a thriving future begins with recognizing planetary health as foundational to human wellbeing and justice.

## Introduction

There is an important connection between the health of people and the health of the planet, both of which can be understood as interacting complex adaptive systems. Health systems require a social-environmental approach that encourages people's agency to function effectively. Similarly, science describes the planet as a complex adaptive system, composed of interconnected bio- and geophysical components—the lithosphere (land), the hydrosphere (water), the biosphere (all living species), the cryosphere (ice), and atmosphere (air and climate), along with their physical, chemical, and biological interactions. According to James Lovelock's Gaia theory (1) the Earth functions like a living entity, self-regulating to remain in a healthy state to buffer and dampen disturbances. To put it simply: the planet must cool to counteract the energy imbalance (i.e., excess heat) created by anthropogenic pressures in the form of greenhouse gas emissions in the last 150 years. If “patient Earth” is left too unattended, it could turn onto an unhealthy path, drifting away from its capacity to self-regulate (i.e., dampen stress). Now, for the first time in human existence on planet Earth, we have ample, unequivocal evidence that patient Earth is in critical condition.

Patient Earth needs regular health checks, and we need to scientifically define a safe operating space on Earth within which equity, prosperity and functional health systems (2) for humans are possible.

## Earth's current health status

The Intergovernmental Panel on Climate Change (IPCC), the International Panel on Biodiversity and Ecosystem Services (IBPES) recognize that there will be no fulfillment of the Sustainable Development Goals without a healthy planet. Actually, there will be no fulfillment of the Paris Climate Agreement unless we're able to take care of the entire Earth system.

Today, we have overwhelming evidence that even if we phase out coal, oil, and gas, i.e., do everything right on phasing out fossil fuels, and achieve a net-zero global economy by 2050, the planet will still drift toward instability. This is because we are failing to safeguard the complex adaptive functions of the Earth system, especially the living components of the biosphere. This is the critical issue we face today.

According to the IPCC, the central message is that not only do we need to phase out fossil fuels, but we also need to ensure that the biosphere on land and the ocean continue to sequester approximately 50% of the carbon dioxide that we emit, to have any chance of delivering on the Paris Climate Agreement (3). On land, this implies that up to 50 % of Earth's surface needs to remain as intact nature. In the ocean this implies that physics, chemistry and biology in marine systems remain within stable ranges of variability.

This is a one scientific example of why we need to define and quantify a “safe operating space” on Earth, for all biophysical systems and processes that regulate the resilience, stability, and the life support systems on Earth (4). The objective? The safeguard human livability on Earth, by upholding the resilience of the entire system, i.e., the ability of Earth to remain in a healthy state via self-regulation.

And what is stability, resilience and life support?

It is health, in a planetary sense. It is the ability of the Earth system to reliably deliver functions that we all, as humanity, depend on. Today we are putting this stability at risk. We have reached a saturation point in terms of human pressures (hitting the ceiling of hard-wired processes that regulate the functioning of the planet).

**Figure 1:**
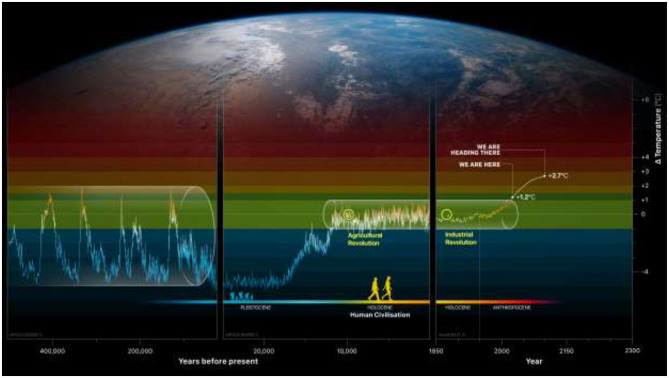
Humanity's Journey on Earth. Global mean surface temperature compared to the pre-industrial average based on Burke et al. (15), Jones et al. (16), Met Office Hadley Centre (17), Marcott et al. (18), Osman et al. (19).

We have also reached a pivotal point in terms of scientific advancement, where we can conclude the following:

1. We have to acknowledge that we are now deep into the Anthropocene, where we, humans in our current world, are the dominating force of change on planet Earth and where risks of irreversible changes are approaching fast.
2. We can today define the health status of the planet. We have a reference point.
3. We know what an unhealthy planet looks like, and we know which symptoms we need to watch out for in the diagnosis (risks of crossing tipping points).
4. Evidence on the reference point for the planet we depend on (i.e., the health status of the planet that we human rely on), is summarized in Figure 1 called Humanity's Journey on Earth.

Figure 1 is an extraordinary scientific advancement—the very basis for the planetary boundary science—which is to understand the evolution of planet Earth over the past 3 million years and humanity's journey on it. On the graph we see the past 400,000 years. Global mean surface temperature rise is currently at 1.2°C above the pre-industrial average, and we are following a pathway that takes us to at least 2.7 by the end of this century, if we follow the current trajectory of environmental degradation and fossil-fuel burning (3). That is a state that planet Earth has not been in for the past 3 million years, and it could put an end to the current geological period, the quaternary. Now, that is number one and it gives us good reason to believe that following a 2.7°C path is highly dangerous, with no evidence that we can provide life support and a chance of a dignified life for eight, soon to be nine billion co-citizens on planet Earth.

However, the most remarkable fact is that since civilizations began to flourish 12,000 years ago, Earth has remained within a remarkably narrow temperature range of 14°C plus minus 0.5°, known as the Holocene. We've had that stability since we left the last Ice Age, 18,000 years ago. Before that, we were at most a few million people on Earth, hunters and gatherers, surviving through two interglacial periods. This way of life stretches back 250,000 years. After leaving the last Ice Age, humanity underwent the Neolithic or agricultural Revolution. We became sedentary farmers, domesticated animals and plants, and began our journey to civilization that ultimately brings us to where we are now.

As far as we understand today, modern humans have only existed on Earth during this 250,000 year period, living through two Ice Ages and two inter-glacials, and for most of it, we've had a very rough time facing massive environmental fluctuations. It wasn't until we entered the Holocene that we landed in an environment of stability and what I call the Garden of Eden, where spring, summer, autumn and winter come back predictably every year. Rainy seasons start and end roughly at the same time, each year. If you're on the savanna, you have 20–25 rainfall events per year, so it's worth investing in planting seed, and having water all the way to harvest. In the temperate zone, you have 15°C in May, which gives you 120 days before you can harvest in September, year in, and year out. Our cultural societies could develop since we had the climatic basis for our modern societies. And we're now propelling ourselves out of this state, recognizing that the Holocene is the reference point for our desired state of the planet.

**Figure 2:**
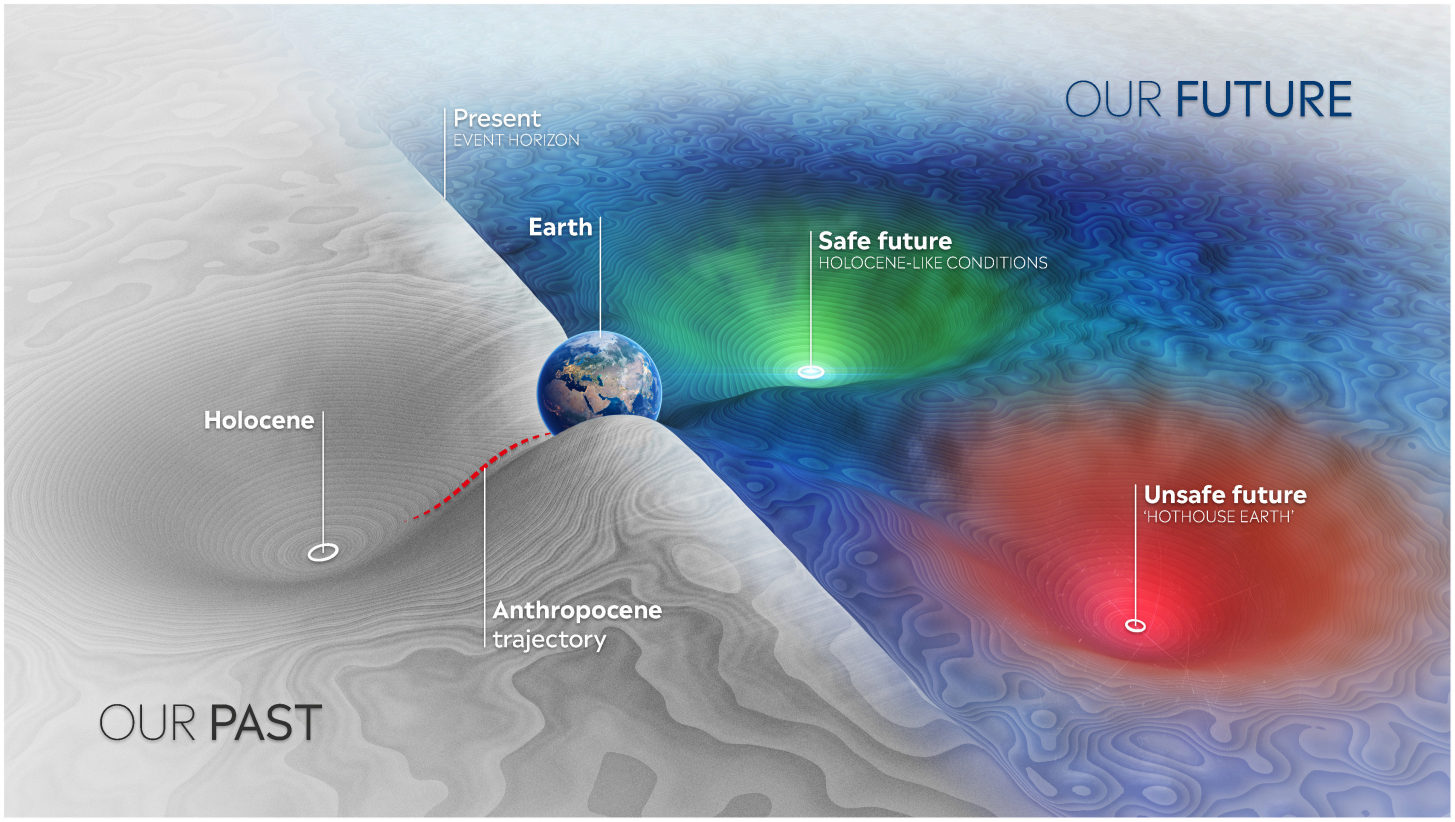
” A stylized picture of the possible states of the Earth system. Currently the Earth is on a path away from the Holocene. Whether it lands in “Holocene-like” conditions, or drifts further away, depends on humanity's efforts to bend the curve on greenhouse gas emissions and protect and restore nature (5).

The question we need to ask is what could push us out of the Holocene? In the Earth Commission, we explored this idea of the Holocene as our “equilibrium attractor” (5). A healthy state of the planet is like a deep cup. In Figure 2, the Earth is represented as a ball in the middle of the cup. We have normal natural variations, including earthquakes, volcanic eruptions, and solar storms, but the strength of the system is such that even if you knock the system, it won't spill over the lip of this cup. Today, we have more and more evidence that the cup itself is getting shallower. It will take less and less to push the Earth system over the cup's edge, which means our planet could tip over to another state, a hothouse Earth state, with a very unsafe future for humanity (6).

## The tipping point

So, back to the question: Why would the planet drift off from the Holocene?

The answer to this is tipping points. When tipping points are crossed, our planet's health deteriorates irreversibly, diminishing its defenses against natural and human-caused disturbances. One could say that tipping elements are the planet's vital organs.

**Figure 3:**
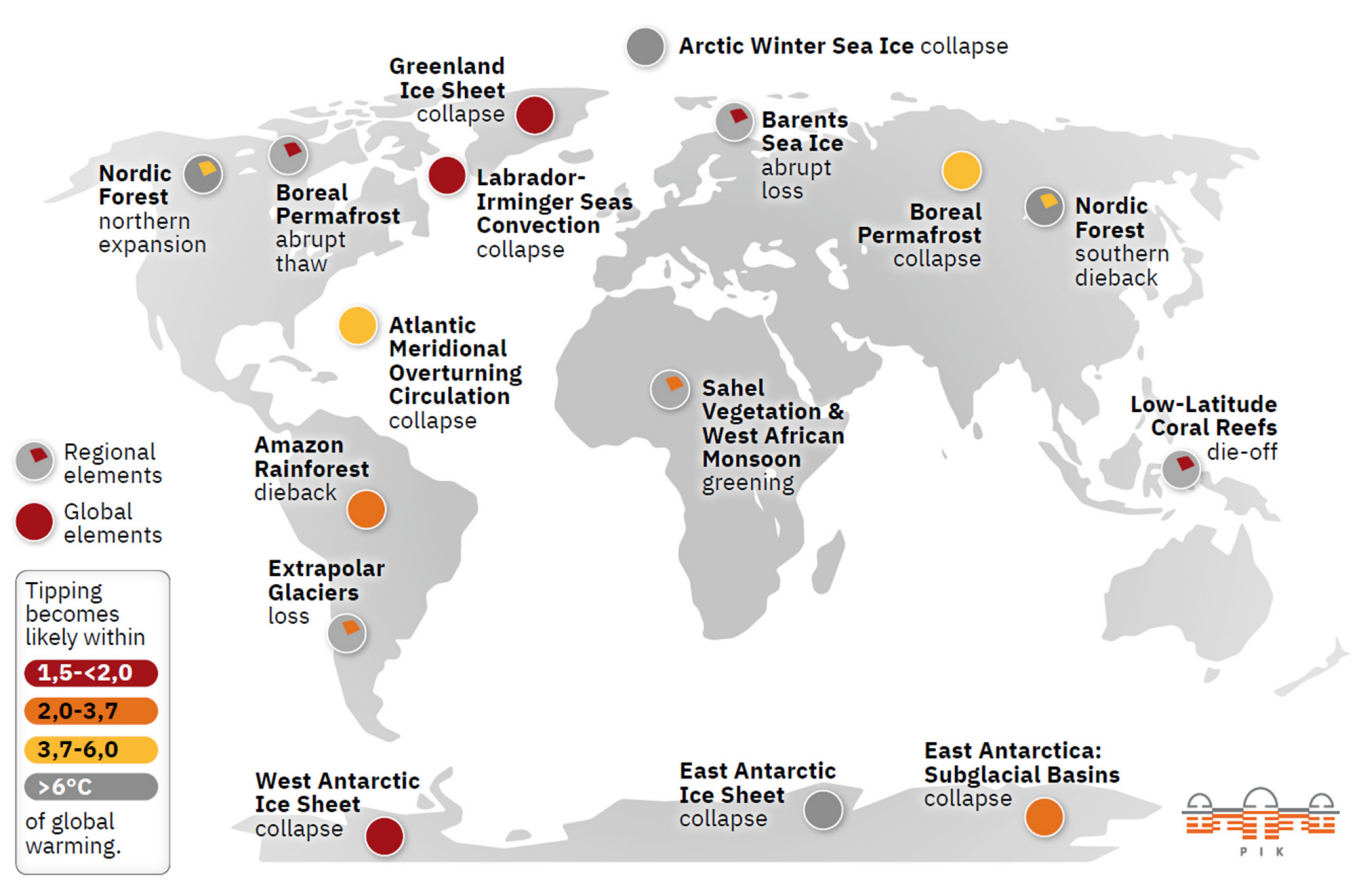
The tipping element system and the global mean temperature rise above which each tipping element is likely to tip (20).

The tipping point system shown in Figure 3, comprising 16 tipping elements, contributes to the regulation of our planet's health. Tipping elements are large-scale components of the Earth that display threshold behavior. Six of them are up in the Arctic with the Greenland Ice Sheet being its mother system. It's connected like an artery through the North Atlantic and the AMOC system. It includes the Amazon Rainforest and connects all the way to the Antarctic Ice Sheet. These are both biological and physical systems with the ocean acting like the arteries and blood of the planet and the biological cycle is embedded into it.

To summarize, the Holocene is the reference point for the desired state of the planet, which we depend on. In addition, we have evidence that the Earth system, is a complex adaptive system (with interactions across all its “spheres”), with a number of biophysical “organs”, so-called tipping elements, that not only contribute to regulate the “health” of the climate system, but also have evidence of multiple stable states, separated by thresholds. Push the system too far, and feedbacks that determine the state of the system will shift, generally from self-dampening (stress) to self-amplifying.

**Figure 4:**
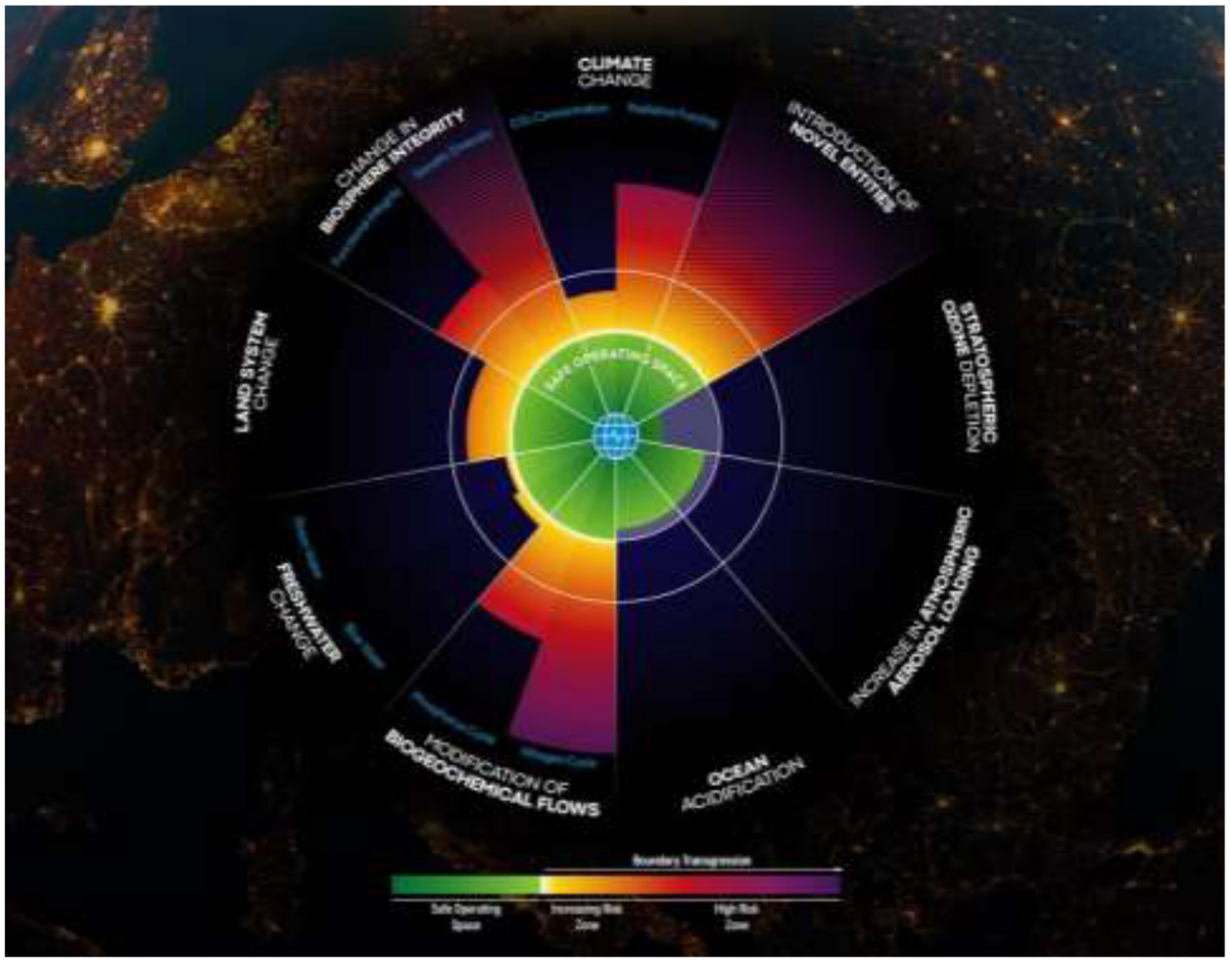
The Planetary Boundary framework, including the current status of each Planetary Boundary (PB Health Check, 2024).

Putting all this evidence together (Anthropocene, Holocene stability, tipping points), has provided science with enough evidence to advance the planetary boundary framework (Figure 4) (7), which aims to answer two key questions:

1. What are the environmental processes that regulate the health of the planet?
2. Once (1) are identified, what are the quantitative boundaries, for each of these processes, beyond which science shows it is likely that the health of the planet can deteriorate in a dangerous way (undermining life-support, resilience and potentially cause irreversible changes)?

These two questions provide the theoretical basis of the Planetary Boundary framework, allowing for the quantification of a safe operating space for humanity on Earth—a definition of Planetary health.

We have identified nine biophysical systems, the nine planetary boundaries: climate change, overloading with novel entities, stratospheric ozone depletion, atmospheric aerosol loading, ocean acidification, interference in biogeochemical flows, freshwater change, land system change and change in biosphere integrity. A very solid example for the interconnection of planetary and human health is air pollution. Air pollution is regulating the big monsoon systems, while air pollution prematurely causes the loss of 7–8 million people per year.

For the first time in 2023 (7) we were able to quantify all these nine boundaries and to place a safe boundary level on each of them. Breaching these safe boundaries means risking a drift away from the healthy state of the planet that we know can support human development on Earth. Six of the nine planetary boundaries have already been breached. The yellow zone marks a zone of uncertainty in science (Figure 4), and we call this the danger zone. Leaving that zone does not necessarily imply the planet would fall over an escarpment, but science points to risks. If we move into the red zone, there is an even higher risk of causing irreversible changes. Climate change is already in the red zone, putting us at high risk. We are facing a climate crisis, and it's not just that biosphere boundaries that are far beyond the safe boundaries. And herein lies an immense double human mistake: We are causing a massive human generated energy imbalance, threatening stability and livability on Earth (8, 9). But instead of preserving a strong and healthy planet, able to buffer that stress, we are undermining that health by moving outside of the safe space on the biosphere boundaries.

Sadly, we now know that Earth's richest biome on terrestrial land, the Amazon rainforest, specifically the south-eastern part, which is also the largest part of the Amazon rainforest, is no longer a carbon sink (21). In fact, it has already shifted to be a net carbon source. This is a warning sign. The Greenland Ice Sheet, too, is melting faster than we had expected (10). It is also important to note that the energy imbalance driving the current climate crisis is mostly absorbed elsewhere. Only 1% of the heat generated by human activities remains in the atmosphere, yet it has already caused a 1.2°C rise in global mean temperature. Ninety percent is taken up by our oceans, 4% is consumed by melting ice, 5% is in the soil (11, 12). We are living on a planet that is doing everything it can to buffer stress, similar to the human body. When a human body is afflicted by stress and disease symptoms, to a certain extent our system tries to buffer against it, which is what our planet is also doing.

Planetary health became a new research field trying to link the physics of Earth system science with the justice dimensions of planetary stability. One example is a recent social science publication from the Earth Commission (13). In this paper, we wanted to define not only the *safe* planetary boundaries, but also the *just* boundaries, by asking ourselves where the limits on the planetary boundaries must be placed to not exceed the maximum tolerable levels of significant harm to people.

And not surprisingly, the safe space shrinks if you take people's health and justice into consideration. The planet may well tolerate more air pollutants than people can. The planet can also tolerate a slightly higher rise in temperature than people can: exceeding 30°C of wet bulb temperatures might be tolerable for some natural systems, but it means life threatening heat levels for humans, endangering wellbeing and life. Today we can already conclude that 2 billion people will be affected by lethal heat at 2°C of warming. Two billion people, who are already living in the most vulnerable areas of the planet, making this a moral and a physical threat to human future.

**Figure 5:**
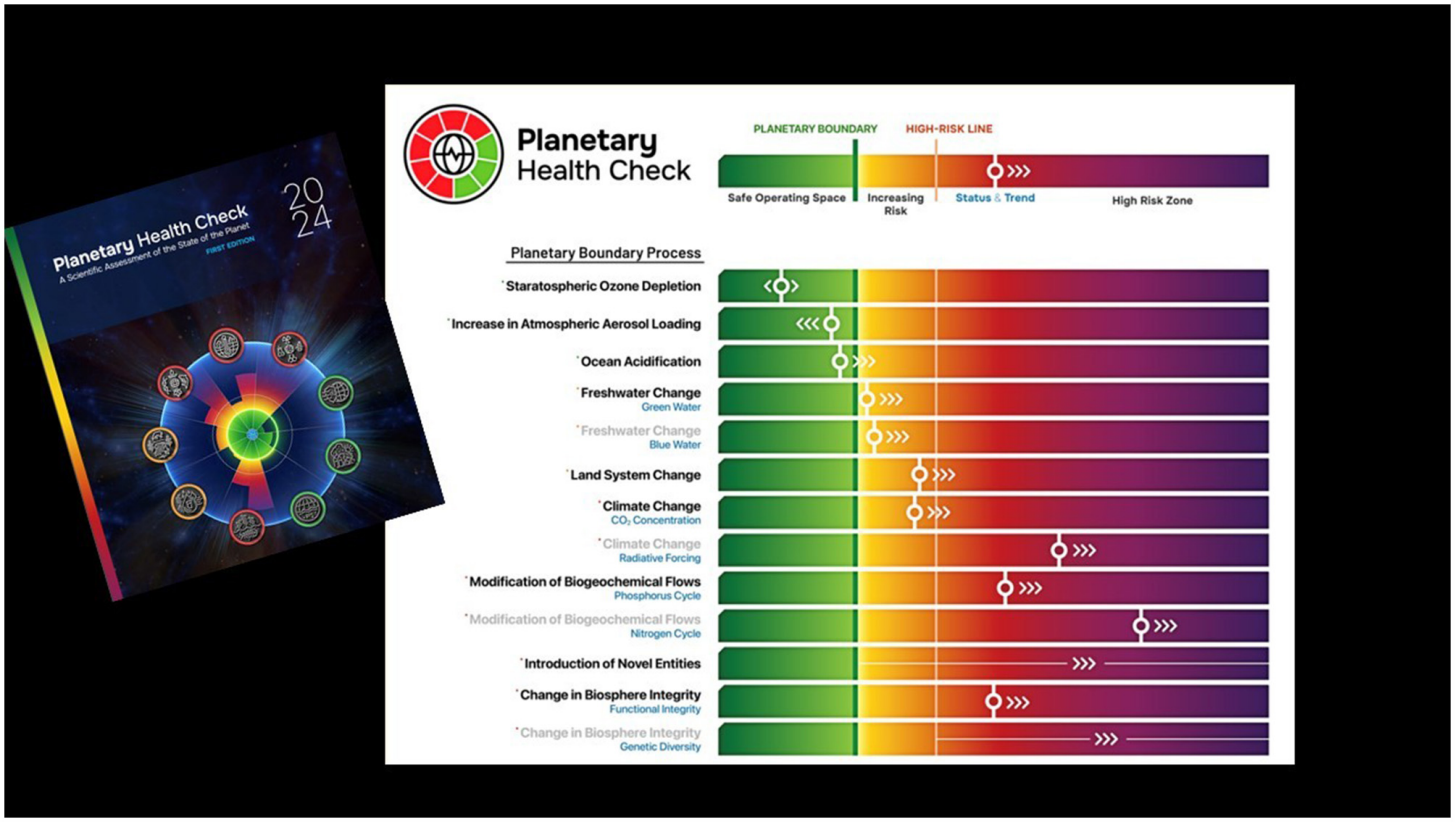
The most up-to-date assessment of the state of the Planetary Boundaries was provided in the 2024 Planetary Boundary Health Check.

In September 2024, during the New York Climate Week, we released the doctor's report for patient Earth by launching the first Planetary Health Check (2) and we are aiming to take planet Earth to the doctor every year for an annual check-up (Figure 5), so it can be thought of as a blood test for the planet.

**Figure 6:**
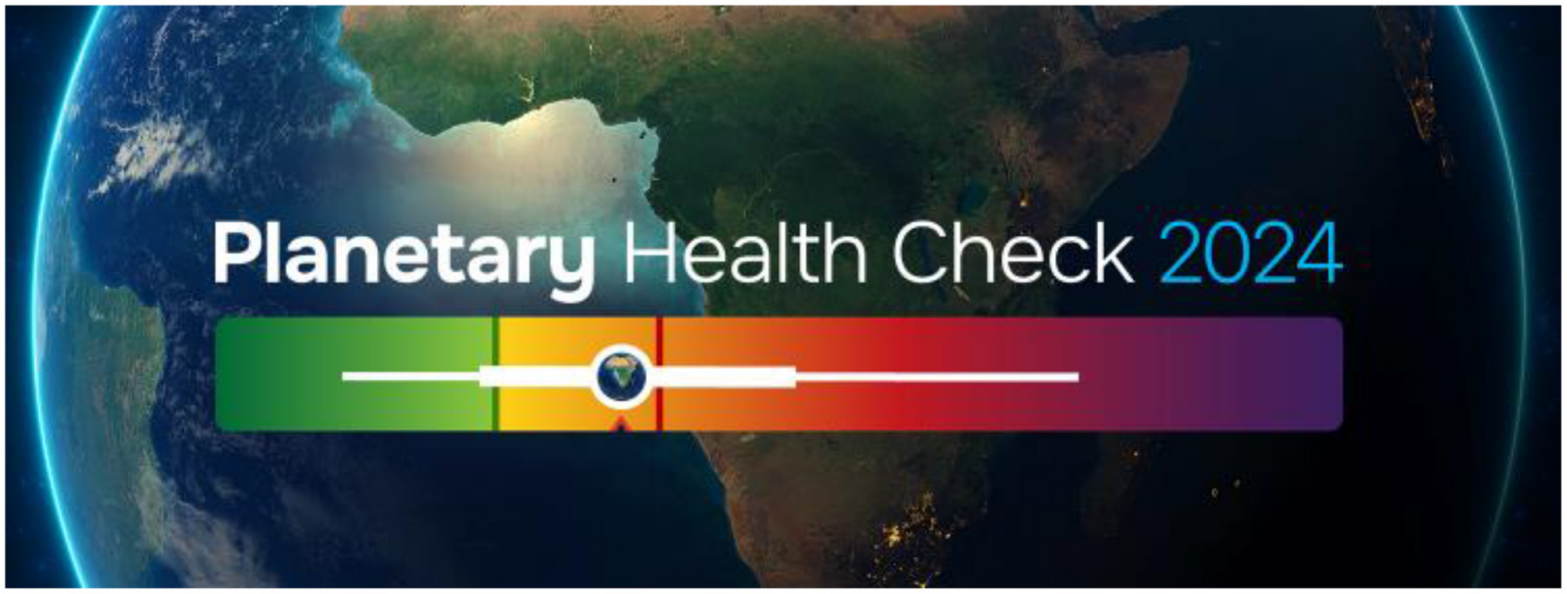
By combining the status of all nine Planetary Boundary into a single diagnostic number, the 2024 Planetary Health Check revealed that the Earth system is currently in the danger zone.

In Figure 5, the safe zone is green, while the yellow to orange zones indicate the uncertainty and danger zones. The white markers cover all nine planetary boundaries with six out of nine boundaries once more confirmed to be outside the safe zone. What is new are the arrows showing that all the boundaries we've already transgressed continue to move in the wrong direction. So not only are we in a danger zone, but we have not even started heading back toward the safe zone. Therefore, we need to urgently shift gear to move back to within the safe space for humanity. We've also tried to summarize this into a single indicator as seen in Figure 6.

The box plot shows the full range of the planetary boundaries and, when intelligently combined, it can be concluded that planet Earth is in the danger zone and very rapidly approaching the red end of the spectrum.

## Conclusion

There is one conclusion I also draw personally. We are in a crisis, but planet Earth is still dominated by negative (dampening, i.e., healthy) feedbacks. It's remarkable that planet Earth, still, under this level of stress, is regulated by cooling and dampening feedbacks. The ocean is still absorbing heat and is still absorbing 25% carbon dioxide (14). Nature on land is still doing everything it can to reduce the heat imbalance we're causing. The window is still open (while barely) for us to have a safe landing. We are not yet in the deep red zone for all the boundaries, but the window is rapidly closing. If we don't bend the curve in the next 5 years on all these boundaries, the chances of a safe and just future for humanity on planet Earth are all but gone.

Humanity is not yet on a transformative journey but getting on track means facing a steep climb. Global emissions need to be reduced by 7.5% per year for the next 10 years (22). We do not have to solve all the problems in the next 5 years, but we have to start transitioning in a new direction. This includes better collaborations between sustainability science and the health community, which is the most powerful lever of change, because that's what touches the heart of every human being.

## Data Availability

The datasets presented in this study can be found in online repositories. The names of the repository/repositories and accession number(s) can be found below: https://www.planetaryhealthcheck.org.
